# Ingestion of 10 grams of whey protein prior to a single bout of resistance exercise does not augment Akt/mTOR pathway signaling compared to carbohydrate

**DOI:** 10.1186/1550-2783-8-18

**Published:** 2011-11-08

**Authors:** Matthew B Cooke, Paul La Bounty, Thomas Buford, Brian Shelmadine, Liz Redd, Geoffrey Hudson, Darryn S Willoughby

**Affiliations:** 1 Department of Health, Human Performance and Recreation, Baylor University, Waco, TX, USA; 2 Institute for Biomedical Studies, Baylor University, Waco, TX, USA; 3 Schools of Medicine and Human Movement Studies, The University of Queensland, Brisbane, QLD, Australia; 4 Department of Aging and Geriatric Research, University of Florida, Gainesville, FL, USA; 5 School of Human Performance and Recreation, University of Southern Mississippi, Hattiesburg

## Abstract

**Background:**

This study examined the effects of a whey protein supplement in conjunction with an acute bout of lower body resistance exercise, in recreationally-active males, on serum insulin and insulin like growth factor 1 (IGF-1) and Akt/mTOR signaling markers indicative of muscle protein synthesis: insulin receptor substrate 1 (IRS-1), AKT, mammalian target of rapamycin (mTOR), p70S6 kinase (p70S6K) and 4E-binding protein 1 (4E-BP1).

**Methods:**

In a randomized, double-blind, cross-over design, 10 males ingested 1 week apart, either 10 g of whey protein (5.25 g EAAs) or carbohydrate (maltodextrose), 30 min prior to a lower-body resistance exercise bout. The resistance exercise bout consisted of 4 sets of 8-10 reps at 80% of the one repetition maximum (RM) on the angled leg press and knee extension exercises. Blood and muscle samples were obtained prior to, and 30 min following supplement ingestion and 15 min and 120 min post-exercise. Serum and muscle data were analyzed using two-way ANOVA.

**Results:**

No significant differences were observed for IGF-1 (p > 0.05). A significant main effect for Test was observed for serum insulin (p < 0.01) at 30 min post-ingestion and 15 and 120 min post-exercise, with no Supplement × Test interaction (p > 0.05). For the Akt/MTOR signaling intermediates, no significant Supplement × Test interactions were observed (p > 0.05). However, significant main effects for Test were observed for phosphorylated concentrations of IRS, mTOR, and p70S6K, as all were elevated at 15 min post-exercise (p < 0.05). Additionally, a significant main effect for Test was noted for 4E-BP1 (p < 0.05), as it was decreased at 15 min post-exercise.

**Conclusion:**

Ingestion of 10 g of whey protein prior to an acute bout of lower body resistance exercise had no significant preferential effect compared to carbohydrate on systemic and cellular signaling markers indicative of muscle protein synthesis in untrained individuals.

## Introduction

The maintenance of skeletal muscle mass is determined by the long-term net balance of skeletal muscle protein synthesis (MPS) and muscle protein breakdown, defined by net protein balance. Though the balance between MPS and muscle protein breakdown is dependent upon feeding state [1-6] as well as training status [7,8], changes in net protein balance are thought to occur predominantly through changes in MPS, which is responsive to both resistance exercise and amino acid provision [9,10]. Resistance exercise leads to acute up-regulation of the inward amino acid transport [11] to the muscle resulting in an elevated fractional synthetic rate of muscle protein for as many as 48 hours following each exercise bout [12].

Some of the principle intracellular signaling pathways involved in MPS are becoming more defined in the literature [13]. As a result, determining the activity of the various pathways, specifically their intermediates, are often used as markers of MPS. MPS is stimulated, at least in part, by the Akt/mTOR pathway, in which pathway intermediate activity is affected by the level of phosphorylation at different amino acid sites [14]. Specifically, the regulation of translation initiation via the Akt/mTOR pathway is recognized as a significant regulator of MPS [15]. Key downstream targets of the kinase mTOR include the eukaryotic initiation factor 4E (eIF4E) binding protein (4E-BP1), which upon phosphorylation releases its inhibition over eIF4E to promote 5'-methylguanosine cap-dependent translation initiation and p70S6 kinase (p70S6K) [16]. Phosphorylation of 4E-BP1 is important due to the fact that it prevents the interaction and inhibition of 4E-BP1 with eIF4E and hence, increases translation and MPS [16]. Conversely, p70S6K influences MPS partially through ribosomal protein S6 (rpS6) as well as through some other proteins such as eukaryotic elongation factor 2 (eEF2) [17].

Ingestion of supplementary protein (whole or as individual amino acids), either before or immediately following resistance exercise training, enhances Akt/mTOR pathway activity and MPS [13,14]. Notwithstanding, ingestion of protein or essential amino acids (EAA) with or without carbohydrate prior to, during, and in the early recovery phase following a bout of resistance exercise can lead to increased phosphorylation of mTOR [15,18], p70S6K [19-21], and rpS6 [22,23] within the first 4 hr post-exercise in both rodent and human models. These results also suggest that timing of ingestion is important, with increased circulation and nutrient transport to the skeletal muscle following exercise occurring concomitantly within the time period when MPS has the greatest elevation in response to exercise [12,24,25]. In addition, protein source and/or dosage appear to play a key role in pre- and post-exercise muscle protein kinetics [26,27]. As little as 10 g of protein (4.2 g EAA) has been shown to stimulate MPS following resistance exercise [27], while acute ingestion of between 20-40 g of intact protein [28], or 9-10 g of EAA [25], seems to induce a plateau in MPS independent of exercise.

Albumin protein intake at a dose of 10 g (4.3 g EAAs) has been shown to significantly increase MPS, but had no effect on the activities of the Akt/mTOR pathway intermediates S6K1 (Thr^389^), rps6 (Ser^240/244^), or eIF2Bε (Ser^539^) after resistance exercise [10]. As a result, we sought to determine if 10 g of whey protein, but with 5.25 g of EAAs, would produce increases in other key Akt/mTOR signalling intermediates following resistance exercise. Therefore, the primary purpose of this study was to determine the consumption of a whey protein supplement prior to an acute bout of lower body resistance exercise in recreationally active males on serum insulin and IGF-1 and the Akt/mTOR signaling markers indicative of MPS: IRS-1, AKT, mTOR, p70S6K and 4E-BP1.

## Methods

### Participants

Ten apparently healthy, recreationally-active (exercise 2-3 times per week), but not resistance-trained (no regular resistance training for at least one year) male participants (20.1 ± 1.4 yrs, 174 ± 8.7 cm, 78.5 ± 12 kg,) participated in this study. All subjects signed informed consent documents and the study was approved by the Baylor University Institutional Review Board for the Protection of Human Subjects prior to any data collection. Subjects were not allowed to participate in this study if they reported any of the following: 1) current or past history of anabolic steroid use; 2) any metabolic disorders or taking any thyroid, hyperlipidmeic, hypoglycemic, anti-hypertensive, or androgenic medications; 3) ingested any ergogenic levels of creatine, HMB, thermogenics, ribose, pro-hormones (i.e., DHEA, androstendione, etc.) or other purported anabolic or ergogenic nutritional supplements within 2 months prior to beginning the study; 4) not taking any additional nutritional supplement or contraindicated prescription medication during the protocol.

### Experimental design

The study was conducted in a cross-over, randomized, double-blinded, and placebo-controlled manner. Participants expressing interest in the study were interviewed on the phone/or via email to determine whether they appear to qualify to participate in this study. Participants believed to meet eligibility criteria were then invited to attend an entry/familiarization session. Once reporting to the lab, participants completed a medical history questionnaire and underwent a general physical examination to determine whether they met eligibility criteria. Once cleared, participants were familiarized to the study protocol via a verbal and written explanation outlining the study design. All eligible participants who agreed to participate in the study read and signed the university-approved informed consent documents. Participants were familiarized with the angled leg press and leg extension machines, the correct technique in performing each of the exercises, and then performed two low-resistance (30% of body mass) practice/warm-up sets of 10 repetitions on each exercise to familiarize them with the protocol and to also insure that they were able to complete the protocol before being formally admitted to the study. Participants then completed an initial strength test to assess their one repetition maximum (1-RM) for each leg on the angled leg press (Nebula Fitness, Inc., Versailles, OH), and leg extension (Body Masters, Inc., Rayne, LA) exercises using standard guidelines routinely employed our laboratory [29]. Following the practice trials, participants were scheduled to return 48 hours later for testing. Participants were asked to not change their dietary habits in any way throughout the study. This was monitored by having each participant document dietary intake for two days before each testing session. In addition, each participant was instructed to fast for eight hours and not to perform any physical activity for the 48 hours proceeding each testing session.

### Resistance exercise protocol

At the beginning of each testing session, participants had their body mass measured according to standard procedures using a self-calibrating digital scale (Health-O-Meter, Bridgeview, IL, USA) with an accuracy of ± 0.02 kg. Participants performed two separate bouts of resistance exercise, each session involving only one leg, each separated by two weeks. The supplement and leg utilized for the first exercise bout was randomly assigned. Using only one leg, participants performed 4 sets of 8-10 repetitions at 75%-80% 1-RM on the angled leg press (Nebula Fitness, Inc., Versailles, OH) and knee extension (Body Masters, Inc., Rayne, LA) exercises. Each set was performed over the course of 25-30 seconds and followed by 120 seconds of rest, while 150 seconds of rest (1:5, work: rest ratio) were allowed between the two exercises. Training volume for each exercise was calculated by multiplying total number of reps by the total amount of weight lifted over the four sets.

### Supplementation protocol

Participants were assigned in a double-blind and randomized manner to orally ingest 10 grams of maltodextrose placebo (CHO) or whey protein (WP) containing 5.25 g of EAAs, mixed with 500 ml of water. Supplements were ingested 30 minutes before each exercise session. Both supplements were isocaloric and independently prepared in individually blinded packages (Glanbia Nutritionals, Twin Falls, ID, USA). The amino acid composition of the WP supplement is displayed in Table 1.

**Table 1:** Amino acid composition of the whey protein (WP) supplement (g/500 ml).

Essential Amino Acids (EAAs)	Concentration (g)
Isoleucine	0.61

Leucine	1.55

Lysine	0.76

Threonine	0.85

Valine	0.63

Methionine	0.32

Tryptophan	0.18

Phenylalanine	0.35

**Total EAAs**	**5.25**

**Non-Essential Amino Acids (NEAAs)**	**Concentration (g)**

Aspartic Acid	0.94

Serine	0.45

Glutamic Acid	1.47

Glycine	0.14

Alanine	0.59

Tyrosine	0.27

Histidine	0.16

Arginine	0.14

Proline	0.44

Cystine	0.15

**Total NEAAs**	**4.75**

**Total Amino Acids**	**10.00**

### Dietary inventories

For two days immediately prior to each testing session, participants were instructed to record all food and fluid intake, which was reflective of their normal dietary intake. Dietary inventories were then analyzed for average energy and macronutrient intake using the ESHA Food Processor Nutritional Analysis software (Salem, OR, USA).

### Blood and muscle collection procedures

Approximately 20 ml of venous blood was obtained from an antecubital vein using standard phlebotomy procedures on four separate occasions at each of the two resistance exercise sessions; 1) 30 min prior to exercise and ingestion of the supplement, 2) immediately before exercise following ingestion of the supplement, 3) 15 min post-exercise, and 4) 120 min post-exercise. Blood analyzed for serum IGF and insulin were placed into two serum separation tubes and immediately centrifuged at 1,100 g for 15 min. Serum was separated and stored at -80°C in polypropylene cryovials for later analysis.

Approximately 50-75 mg of muscle was obtained from the lateral portion of the *vastus lateralis *midway between the patella and iliac crest of the leg using a 5-mm Bergstrom style biopsy needle. Muscle samples were taken on 3 separate occasions at each of the two resistance exercise sessions; 1) 30 min prior to exercise and ingestion of the supplement, 2) 15 min post-exercise, and 3) 120 min post-exercise. Participants were instructed to refrain from exercise 48 hr prior to each muscle biopsy. After removal, adipose tissue was trimmed from the muscle specimens and immediately frozen in liquid nitrogen and then stored at -80°C for later analysis.

### Serum IGF and insulin

The concentrations of serum insulin and IGF-1 were determined in duplicate and the average concentrations reported using commercially available enzyme-linked immunoabsorbent assay (ELISA) kits (Diagnostic Systems Laboratories, Webster, TX; Biosource, Camarillo, CA). Standard curves were generated using specific control peptides. Concentrations were determined at an optical density of 450 nm with a microplate reader (Wallac Victor 1420, Perkin Elmer, Boston, MA, USA). The overall intra-assay percent coefficient of variation was 4.6% and 2.9% for insulin and IGF-1, respectively.

### IRS-1 and Akt/mTOR signaling pathway protein expression

Approximately 20 mg of each muscle sample was homogenized using a commercial cell extraction buffer (Biosource, Camarillo, CA, USA) and a tissue homogenizer. The cell extraction buffer was supplemented with 1 mM phenylmethanesulphonylfluoride (PMSF) and a protease inhibitor cocktail (Sigma Chemical Company, St. Louis, MO, USA) with broad specificity for the inhibition of serine, cysteine, and metallo-proteases. Muscle homogenates were analyzed for phosphorylated IRS-1 (Ser312), Akt (Ser473), 4E-BP1 (Thr46) and p70S6K (Thr389) using commercially-available phosphoELISA kits (Invitrogen, Carlsbad, CA, USA). This sensitivity of these particular assays is reported by the manufacturer to be less than 1 U⁄mL. The absorbances, which are directly proportional to the concentration in the samples, were determined at 450 nm with a microplate reader (Wallac Victor 1420, Perkin Elmer, Boston MA, USA). A set of standards of known concentrations for each phosphorylated muscle variable were utilized to construct standard curves by plotting the net absorbance values of the standards against their respective protein concentrations. By applying a four part parameter curve using MikroWin microplate data reduction software (Microtek Lab Systems, Germany), the concentrations in the muscle samples were appropriately calculated. Protein concentrations were expressed relative to muscle wet-weight. The overall intra-assay percent coefficient of variation for all assays was less than 7%

Phosphorylated mTOR was assessed through the use of ELISA used by methods previously described [29]. A polyclonal antibody specific for phosphorylated mTOR (Ser 2448), where target antigen specificities have been verified through Western blotting by the manufacturer, was purchased from Santa Cruz Biotech (Santa Cruz, CA). Initially, the antibody was diluted to 0.5 μg/ml in coating buffer (Na2CO3, NaHCO3, and ddH2O, pH 9.6) and allowed to incubate at room temperature overnight. Following incubation, the plates were washed (1 × phosphate buffered saline, Tween-20), blocked (10 × phosphate buffered saline, bovine serum albumin, ddH2O), washed, and then incubated with a secondary antibody (IgG conjugated to HRP) diluted to 0.5 μg/ml in dilution buffer (10 × phosphate buffered saline, Tween-20, bovine serum albumin, ddH2O). After washing, a stabilized TMB chromogen was added and the plates were covered and placed in the dark for the last 30-min prior to being stopped with 0.2 M sulphuric acid. The subsequent absorbances, which are directly proportional to the concentration of the phosphorlyated mTOR in the samples, were measured at a wavelength of 450 nm. There were no standards used in this ELISA, thus no standard curve was created. Therefore, the absorbances relative to muscle weight were assessed. The overall intra-assay percent coefficient of variation was 7.12%.

### Statistical analyses

Data are presented in all tables and throughout the text as mean ± SD. Serum IGF and insulin were analyzed using 2 × 4 [Supplement (CHO, WP) × Test (pre, 30 min post supp, 15 min post-ex, and 120 min post-ex)] factorial analyses of variance (ANOVA) with repeated measures on the Test factor. Muscle protein levels were analyzed using 2 × 3 [Supplement (CHO, WP) × Test (pre, 15 min post-ex, and 120 min post-ex)] factorial ANOVA with repeated measures on the Test factor. Further analysis of the main effects was performed by separate one-way ANOVAs. Significant between-group differences were determined using Bonferroni Post-Hoc Test. Participant characteristics, resistance exercise volume, and 1-RMs for the angled leg press and leg extension exercises for each testing session were analyzed using a paired sample t-test. All statistical procedures were performed using SPSS 16.0 software and a probability level of p < 0.05 was adopted throughout.

## Results

### Participant characteristics and supplement side effects

There were no significant differences in the body weight, resting blood pressure, or heart rate between the two testing sessions (data not shown). In a post-study questionnaire administered in a blinded manner, no adverse events were reported concerning the supplementation or study protocol.

### Dietary analysis

Analysis of dietary intake (excluding supplementation) for two days immediately prior to each testing session revealed no differences (p > 0.05) in total caloric, protein, fat, or carbohydrate intake between testing session during the course of the study (Table 2).

**Table 2:** Dietary analyses performed two days immediately prior to each testing session.

Dietary Variable	WP	CHO	p-value
Total Calories (kcal/kg/day)	31.14 ± 7.3	30.43 ± 5.1	0.84

Protein (g/kg/day)	0.83 ± 0.2	0.86 ± 0.1	0.73

Fat (g/kg/day)	0.93 ± 0.1	0.96 ± 0.1	0.22

Carbohydrate (g/kg/day)	4.40 ± 0.9	4.22 ± 1.32	0.13

Data are means ± standard deviations. SI unit conversion factor: 1 kcal = 4.2 kJ. Values exclude supplementation dose.

### Muscle strength and resistance exercise volume

There were no significant differences in the 1-RM values between legs at each testing session for the angled leg press (p = 0.35) and leg extension (p = 0.42) exercises. The 1-RM for the leg press was 156.05 ± 18.86 kg for the right leg and 154.29 ± 25.52 kg for the left leg, and the 1-RM for the leg extension was 44.94 ± 3.91 kg for the right leg and 44.69 ± 5.11 kg for the left leg. Additionally, there were no significant differences in the resistance exercise volume between the two testing sessions. The volume for leg press was 4744.5 ± 960.4 kg for WP and 4841.6 ± 1212.9 kg for CHO (p = 0.89), and the volume for leg extension was 1187.5 ± 267.6 kg for WP and 1285.2 ± 180.1 kg for CHO (p = 0.35).

### Serum IGF-1 and insulin

For IGF-1, no significant main effects for Supplement and Test or the Supplement × Test interaction were observed (p > 0.05) (Table 3). For insulin, no significant main effect for Supplement or the Supplement × Test interaction was observed (p > 0.05); although, a significant main effect for Test (p < 0.001) was observed. Post-hoc analysis showed significant differences between baseline, 30 min post-supplement ingestion, 15 min post-exercise, and 120 min post-exercise (Table 3).

**Table 3:** Serum IGF-1 and insulin levels for WP and CHO.

Variable	Time Point	WP	CHO	p-value
IGF-1 (ng/ml)	Baseline	0.46 ± 0.4	0.39 ± 0.3	Supplement (S) = 0.64

	30 min post-ingestion	0.47 ± 0.4	0.45 ± 0.4	Test (T) = 0.34

	15 min post-exercise	0.44 ± 0.5	0.39 ± 0.3	S × T = 0.89

	120 min post-exercise	0.50 ± 0.4	0.44 ± 0.3	

Insulin (μIU/ml)	Baseline	12.83 ± 6.1	14.05 ± 7.1	Supplement (S) = 0.95

	30 min post-ingestion	51.90 ± 25.3	50.59 ± 34.9	Test (T) = 0.001†¥#

	15 min post-exercise	23.60 ± 14.1	14.62 ± 8.9	S × T = 0.76

	120 min post-exercise	10.08 ± 6.5	9.33 ± 5.5	

Data are means ± standard deviations.

† represents significant difference from baseline at 30 min post-ingestion.

¥ represents significant difference from baseline at 15 min post-exercise.

# represents significant difference from baseline at 120 min post-exercise.

### Akt/mTOR signaling intermediates

While no significant main effects for Supplement or the Supplement × Test interaction were observed for any of the variables (p > 0.05), a significant main effect for Test (p < 0.05) was observed for IRS-1 (p = 0.040), mTOR (p = 0.002), p70S6K (p = 0.046), and 4E-BP1 (p = 0.001). No significant main effects for Test was observed for Akt (p = 0.359). Subsequent analyses revealed a significant increase from baseline in IRS-1 at 15 and 120 m post-exercise, an increase in mTOR and p70S6K at 15 min post-exercise, and a significant decrease in 4E-BP1 at 15 min post-exercise (Table 4).

**Table 4:** Phosphorylated levels of Akt/mTOR pathway intermediates for WP and CHO.

Variable	Time Point	WP	CHO	p-value
IRS-1	Baseline	15.68 ± 9.6	19.52 ± 6.4	Supplement (S) = 0.88

	15 min post-exercise	29.04 ± 6.6†	22.28 ± 11.2	Test (T) = 0.04†#

	120 min post-exercise	25.40 ± 6.0	19.65 ± 9.2	S × T = 0.44

Akt	Baseline	5.04 ± 1.9	6.88 ± 1.1	Supplement (S) = 0.21

	15 min post-exercise	6.04 ± 2.6	5.61 ± 4.1	Test (T) = 0.35

	120 min post-exercise	4.78 ± 1.4	4.58 ± 2.1	S × T = 0.82

mTOR	Baseline	3.34 ± 0.34	3.62 ± 0.19	Supplement (S) = 0.93

	15 min post-exercise	3.75 ± 0.62	3.66 ± 0.27	Test (T) = 0.002†

	120 min post-exercise	3.33 ± 0.19	3.52 ± 0.28	S × T = 0.34

P70S6K	Baseline	8.51 ± 3.2	10.41 ± 3.2	Supplement (S) = 0.96

	15 min post-exercise	14.14 ± 6.6	11.18 ± 2.9	Test (T) = 0.04

	120 min post-exercise	13.32 ± 6.1	11.24 ± 5.0	S × T = 0.74

4E-BP1	Baseline	4.30 ± 2.4	5.33 ± 1.7	Supplement (S) = 0.28

	15 min post-exercise	2.66 ± 1.3†	2.28 ± 1.0	Test (T) = 0.001†

	120 min post-exercise	4.07 ± 1.9#	4.90 ± 1.8	S × T = 0.64

Data are means ± standard deviations.

p70S6K, eIF4E-BP1, AKT and IRS-1 are expressed as U/ml/mg.

mTOR is expressed as absorbance units at 450 nm/mg.

† represents significant difference from baseline at 15 min post-exercise.

# represents significant difference from baseline at 120 min post-exercise.

## Discussion

In the present study, we chose to assess changes in the activity of Akt/mTOR pathway intermediates as markers of MPS in response to resistance exercise after ingesting 10 g of whey protein. As a result, we observed resistance exercise to effectively activate signaling intermediates of the Akt/mTOR pathway. Specifically, we demonstrated increased phosphorylation of IRS-1, AKT, and mTOR. Relative to their downstream targets, p70S6K was hyper-phosphorylated at 15 min post-exercise, whereas 4E-BP1 was hypo-phosphorylated at 15 min post-exercise. Conversely, we also observed that ingesting 10 g of whey protein was unable to induce a greater response in such kinase phosphorylation when compared to ingesting carbohydrate. Therefore, our results suggest that ingestion of 10 g of whey protein (5.25 g EAAs) is no different than an equal amount of carbohydrate at enhancing the activity of systemic and cellular signaling markers indicative of MPS following resistance exercise.

Resistance exercise and amino acids effectively stimulate MPS [30]. Based on previous studies, the role that nutrient ingestion plays in activating the Akt/mTOR pathway [15,18-20] is not completely understood, and may likely be related to the amount of amino acids available or whether co-ingested with carbohydrate. Previous studies have demonstrated that 20 g of whey protein (8.6 g EAAs) [10] and 10 g EAAs [26] maximally stimulated MPS, but that MPS was also increased even at whey protein doses of 5 g (2.2 g EAAs) and 10 g (4.3 g EAAs) [10] and an EAA dose of 5 g [26]. When smaller amounts of EAAs (3-6 g) were ingested, with [31] and without [32] carbohydrate, the post-exercise increase in MPS was similar, but greater than basal or post-exercise fasted levels. In the present study, rather than assessing MPS, our interest was primarily focused on the extent with which 10 g of whey protein comprised of 5.25 EAAs would affect the activity of the Akt/mTOR pathway after resistance exercise when compared to carbohydrate alone and if this activity might also be systemically affected by either insulin or IGF-1. The reason for our interest was an attempt to discern if the 5.25 g of EAAs contained within 10 g of whey protein, without carbohydrate, was adequate to activate the Akt/mTOR compared to carbohydrate in response to a single bout of resistance exercise. Our interest was heightened by a previous study in which albumin protein intake at 10 g (4.3 g EAAs) significantly increased MPS, and maximally when 20 g (8.6 g EAAs) and 40 g (16.4 g EAAs) were ingested, yet none of the three concentrations had any affect on the activities of the Akt/mTOR pathway intermediates S6K1 (Thr^389^), rps6 (Ser^240/244^), or eIF2Bε (Ser^539^) at 60 and 240 min post-exercise [10]. Despite previous evidence indicating otherwise [10], we were curious to determine if 10 g of whey protein would produce increases in other key Akt/mTOR signalling intermediates following resistance exercise.

It is evident that acute resistance exercise results in a significant increase in the rate of initiation of protein synthesis compared with resting muscle [33]. It is suggested that signal transduction pathways control the rate of initiation of MPS, and appear to be the key factors in the hypertrophic process [34,35]. Of particular importance is the complex myriad of signaling proteins, with Akt suggested to be a key regulator. Maximal activation of Akt occurs through phosphorylation of Ser473 and it appears that Akt may have a relatively short period of activation after an acute bout of resistance exercise [36]. Research into the regulation of Akt signalling by exercise has produced conflicting results. A series of studies have demonstrated that contractile activity either positively or negatively regulates Akt activity [15,37-39], while others failed to find any change [40-42]. In the current study, we found that resistance exercise and nutrient ingestion failed to induce a significant change in the phosphorylation of Akt.

Stimuli of the Akt pathway includes hormones and muscle contraction. Insulin [43] and IGF-I [44] bind to their respective membrane-bound receptors and subsequently activate phosphatidylinositol-3 kinase (PI-3K), an upstream activator for Akt phosphorylation. Quantification of circulating IGF-I levels has yielded inconsistent results, with levels being reported to decline [45], increase [46], or remain unchanged [47] after the onset of exercise. Furthermore, circulating IGF-1 has been shown to have no direct effect on muscle hypertrophy [48]. In the current study, we observed no changes in serum IGF-1 following the exercise bout or due to nutrient ingestion, thereby suggesting hepatically-derived IGF-1 to have no appreciable effect on Akt pathway activation.

Serum insulin was increased in both groups. It is evident as to why insulin increased in the CHO group as 10 g of carbohydrate were ingested. In addition, the WP group also underwent a similar increase in insulin in the absence of ingested carbohydrate, which is in agreement with the insulin response previously demonstrated with 20 g of whey protein (10 g EAAs) [49]. The Akt/mTOR signalling pathway is activated by insulin. Insulin binds with its receptor and leads to an increase in tyrosine phosphorylation of IRS-1 and eventually mTOR activation. In the present study, insulin significantly increased in both groups 30 min post-supplement ingestion and 15 min post-exercise, which was mirrored by significant increases in IRS-1 activation at 15 min post-exercise. Even though Akt phosphorylation was not significantly increased, activation of IRS-1 likely contributed to the observed increases in mTOR activation; however, this activity was not preferentially contingent on 10 g of whey protein ingestion.

mTOR is a 289 kDa serine/threonine kinase downstream of Akt and stimulates protein synthesis through downstream activation of p70S6K and 4E-BP1, providing a key point of convergence for both resistance exercise and amino acids [14]. Amino acid ingestion has been shown to significantly enhance mTOR signalling [25,50]. In the present study, the acute bouts of resistance exercise significantly increased mTOR and p70S6K activation at 15 min post-exercise, while a marked decrease in 4E-BP1 activation was also observed at 15 min post-exercise. While we observed mTOR activation to be enhanced by resistance exercise, the Akt/mTOR pathway signalling intermediates we assessed were unaffected by the provision of 10 g of whey protein comprised of 5.25 g EAAs.

Previous work has suggested that a minimal amount of 20 g is needed to stimulate MPS [10]; however, others have demonstrated positive effects utilizing a dosage as low as 6 g EAAs [51]. Increases in MPS following resistance exercise have been observed when utilizing 10 g of whey protein; however, the protein supplement was co-ingested with 21 g of carbohydrate [26]. However, it has recently been shown that approximately 5 g (2.2 g EAAs) and 10 g (4.2 g EAAs) of whey protein without carbohydrate significantly increased MPS 37% and 56%, respectively, over baseline. In this study, it was also shown that 20 g (8.6 g EAAs) maximally stimulated MPS following resistance exercise [27]. Although, our results are supported by previous data which demonstrated that 20 g of albumin protein (8.6 g EAAs) enhanced MPS after resistance exercise, yet had no effects on activation of the mTOR pathway intermediates, S6K1, rps6, and eIF2Bε post-exercise [27], the dosage used in the current study (10 g whey protein, 5.25 g EAA) was apparently insufficient to reach an amino acid dosage capable of stimulating Akt/mTOR pathway activity.

A number of limitations exist in the current study. Firstly, we only assessed the relative changes in the phosphorlated levels of various Akt/mTOR pathway intermediates. Thus, these can only be used as markers indicative of MPS. We did not measure protein synthesis directly and thus caution needs to be taken when interpreting changes in phosphorylation status of signaling pathway intermediates to imply changes in human MPS, as this does not always determine functional changes. Secondly, no control was used and thus no direct comparison between isoenergetic carbohydrate and whey protein and resistance exercise could be made. However, previous research has clearly indicated that resistance exercise robustly activates Akt/mTOR signalling. Thirdly, only one dosage was used (10 g) and thus any comparison between other dosages cannot be made directly. Finally, our study focused on the early post-exercise recovery response in signalling and, therefore, we acknowledge the possibility that long-term activation of Akt/mTOR signalling and its downstream targets such as at 6, 24, or 48 hr post-exercise may be better indicators of muscle MPS over the course of a resistance training program.

In conclusion, the present study shows that ingestion of 10 g whey protein (5.25 g EAAs) prior to a single bout of lower body resistance exercise had no significant effect on activating systemic and cellular signaling intermediates of the Akt/mTOR pathway, otherwise indicative of MPS, in untrained men. Future research should examine the effects of dose response and timing of protein ingestion and compare the effects of various forms/fractions of proteins on post-exercise cell signalling responses to resistance exercise.

## Competing interests

All researchers involved independently collected, analyzed, and interpreted the results from this study and have no financial interests concerning the outcome of this investigation.

## Authors' contributions

MC coordinated the study, carried out the exercise sessions and all analyses, and drafted the manuscript. PLB carried out the exercise sessions and helped with analysis. TB helped with the biochemical analysis LR helped with exercise testing sessions BS helped with exercise sessions biochemical analysis GH helped with exercise sessions biochemical analysis. DSW conceived the study, developed the study design, secured the funding for the project, assisted and provided oversight for all data acquisition and statistical analysis, assisted and provided oversight in drafting the manuscript, and served as the faculty mentor and principal investigator for the project. All authors read and approved the final manuscript.
